# Impact of sampling technique, anticoagulant, processing delay, and temperature on murine platelet function in whole blood

**DOI:** 10.1016/j.rpth.2025.102883

**Published:** 2025-05-08

**Authors:** Silvia Maria Grazia Trivigno, Alice Assinger, Waltraud Cornelia Schrottmaier

**Affiliations:** 1 Institute of Vascular Biology and Thrombosis Research, Centre of Physiology and Pharmacology, Medical University of Vienna, Vienna, Austria; 2 University School for Advanced Studies, Istituto Universitario di Studi Superiori (IUSS), Pavia, Italy; 3 Department of Biology and Biotechnology, University of Pavia, Pavia, Italy

**Keywords:** animal experimentation, anticoagulant, blood specimen collection, delayed processing, method optimization, platelet activation, specimen handling, temperature

## Abstract

**Background:**

Platelets are highly sensitive to subtle changes in their microenvironment, making functional analyses challenging and prone to variation. Advances in understanding how experimental procedures influence human platelet activation have improved the accuracy and comparability of diagnostic and research data. However, despite the pivotal role of murine models, the effects of methodological variations on murine platelets remain incompletely understood.

**Objectives:**

To elucidate how blood draw techniques, anticoagulation, processing delay, and assay temperature affect murine platelets.

**Methods:**

Blood was obtained by retro-orbital, vena cava, or cardiac puncture and anticoagulated with heparin, citrate, or acid-citrate-dextrose ± recalcification. After 30 to 120 minutes, blood was stimulated at room temperature or 37 °C with adenosine diphosphate (ADP), protease-activated receptor 4-activating peptide (PAR4-AP), or cross-linked collagen-related peptide (CRP-XL), and platelets were analyzed by flow cytometry for CD62P, CD63, CD40L, and activated glycoprotein IIb/IIIa.

**Results:**

Blood sampling had minimal impact on ADP-induced platelet activation. However, platelets isolated via vena cava or cardiac puncture exhibited heightened responsiveness to PAR4-AP and CRP-XL, respectively, compared with retro-orbital sampling. Citrate and acid-citrate-dextrose significantly impaired PAR4-AP responses compared with heparin, whereas CRP-XL sensitivity was anticoagulant-independent. Processing delays as brief as 60 minutes significantly altered platelet reactivity to CRP-XL and PAR4-AP, with further delays producing minimal additional impact. Finally, ADP- and CRP-XL–induced platelet activation was significantly reduced at 37 °C compared with room temperature.

**Conclusion:**

Common variations in murine platelet handling influence *in vitro* responsiveness of platelets in an agonist-specific manner, highlighting the critical need for meticulous assay optimization to ensure experimental consistency and comparability.

## Essentials

• Common protocol variations in murine platelet handling may influence platelet sensitivity.
• Effects of blood draw technique, anticoagulant, processing delay, and temperature were examined.
• Protocol variables affected murine platelet responsiveness in an agonist-specific way.
• Meticulous assay optimization is crucial to ensure comparability between experiments and studies.

## Graphical abstract

**Figure:**
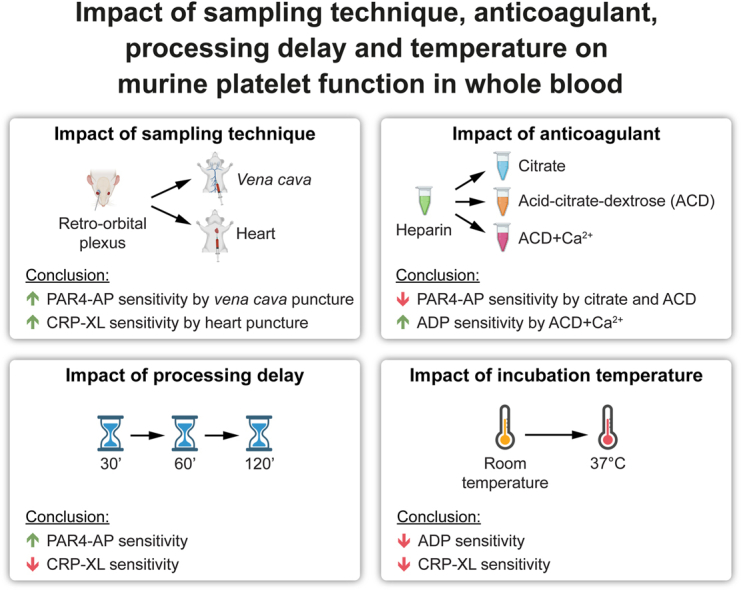

## 1 Introduction

Platelets are central players in hemostasis and (immuno-)thrombosis, and their proper function is crucial to maintain vascular integrity, prevent bleeding, and avert thrombotic events [1,2]. Due to the highly sensitive nature of platelets and their use of positive feedback loops to enhance activation, even minor changes in the microenvironment can significantly impact their functional responses. In recent years, human platelets have been demonstrated to respond to mechanical, chemical, and thermal stimuli such as acoustic vibration [3], pneumatic transport [4], anticoagulation [5,6], pH [7,8], and temperature [[9], [10], [11]]. Accordingly, standardization guidelines for drawing and handling human blood in clinical settings aim to ensure safety, diagnostic validity, and accuracy [12], and aspirations to harmonize assay procedures are increasingly finding their way into research communities as well [13,14].

Murine models have proven instrumental in exploring the genetic, cellular, and molecular mechanisms underlying hemostatic disorders and have pivotally contributed to our understanding of aberrant platelet function, eg, during vascular disease, obesity, autoimmunity, or sepsis, thereby aiding in the inception and development of new therapy strategies [[15], [16], [17], [18], [19], [20]]. However, the impact of microenvironmental changes on murine platelets is still poorly understood. Although murine and human platelets share a highly conserved proteome and exhibit similar biophysical parameters [21,22], the expression patterns of murine platelets also differ from their human counterparts regarding several key receptors, signaling enzymes, and cytoskeletal proteins, such as protease-activated receptor (PAR) and purinergic receptors, protein kinase C isoforms, and talin [[23], [24], [25], [26]].

Therefore, findings in murine models should not be indiscriminately extrapolated to human systems, and *vice versa*. Experimental procedures in murine models, including blood drawing, anticoagulation, and incubation temperatures, often vary between groups. While there are 2 prevailing approaches regarding assay temperature—room temperature (RT) vs physiological 37 °C—applied at the researcher’s discretion, blood sampling techniques are constrained by local and national ethical guidelines and thus may not be interchangeably utilized. Furthermore, specific experimental constraints, such as required blood volumes (that may dictate the choice of blood sampling site) or the need to process large cohorts (resulting in variable processing times for individual samples), can introduce protocol variations even within the same laboratory or experiment. Understanding the microenvironmental regulation of murine platelet function is thus essential for designing experiments that minimize bias between groups. This study aimed to elucidate how common procedural variations—processing delay, blood draw technique, anticoagulation, and temperature—influence the *in vitro* responsiveness of murine platelets to different prothrombotic agonists. Using glycoprotein (GP) IIb/IIIa activation and degranulation markers CD62P, CD63, and CD40L as readouts, we observed that while activation markers were generally influenced in a similar manner by protocol modifications, the specific effects of these variations differed depending on the agonist used.

## 2 Methods

### 2.1 Animals

C57BL/6J mice were maintained at the animal facilities of the Medical University of Vienna (Austria) and housed in same-sex groups in plastic cages under controlled conditions: 20 to 24 °C; 45% to 65% humidity; a 12:12 light/dark cycle; and free access to food and water. Experiments were approved by the Animal Care and Use Committee of the Medical University of Vienna and the Austrian Ministry of Sciences (BMBWF-2024-0.019.492) in accordance with Animal Research: Reporting of *In Vivo* Experiments (ARRIVE) guidelines and the European Union Directive 2010/63 for the protection of animals used for scientific purposes. Both male and female mice, aged 12 to 30 weeks, were used for experiments, with equivalent male:female ratios in experimental groups that were directly compared.

### 2.2 Blood collection

Mice were anesthetized with isoflurane (Forane; Baxter Healthcare Corporation) for retro-orbital (RO) puncture or, when comparing different sampling sites with intraperitoneal injection of an overdose of ketamine (250 μg/g; Ketasol; Livisto) and xylazine (25 μg/g; Xylasol; AniMedica) for RO, vena cava (VC), or heart (H) puncture. Analgesia was tested by checking for pain response via deep toe pinch. After blood sampling, mice were euthanized by cervical dislocation.

#### 2.2.1 RO puncture

The RO plexus was punctured using heparinized microhematocrit capillaries (Brand). Blood was drawn through the capillary into plastic tubes filled with 60 μL of anticoagulant solution until a final volume of 300 μL.

#### 2.2.2 VC puncture

The anesthetized mice were fixed in a dorsal position, the abdomen was opened, and the VC was exposed. A 1 mL syringe with a 27-G needle was prepared with 100 μL of anticoagulant prepositioned within the needle’s dead volume. The VC was punctured, and 500 μL of blood was slowly aspirated.

#### 2.2.3 H puncture

The anesthetized mice were fixed in a dorsal recumbency. A 1 mL syringe with a 27-G needle was prepared with 100 μL of anticoagulant prepositioned in the dead volume. The bevel of the needle was placed up and advanced just to the left of the animal’s xiphoid, under the ribs at a flat angle of roughly 20° to 30°. During entry, the plunger was slightly retracted, and 0.5 cm^3^ of air was withdrawn to create a vacuum in the syringe. Upon reaching the H, blood immediately appeared in the syringe, and 500 μL was slowly aspirated to avoid the H collapsing.

### 2.3 Anticoagulants

For standard blood sampling, heparin (Biochrom) was used at a final concentration of 25 U/mL. For experiments investigating the effect of calcium-chelating anticoagulants, acid-citrate-dextrose (ACD; Sigma-Aldrich) or 3.2% sodium citrate (Greiner) was added to heparinized whole blood in a ratio of 1:10. ACD-anticoagulated blood was optionally recalcified by adding CaCl_2_ to a final concentration of 2 mM. Whole blood pH was measured by pH meter using a suitable pH electrode (InLab Micro, Mettler Toledo).

### 2.4 Platelet activation

Anticoagulated whole blood samples were activated at RT (22-26 °C) or 37 °C for 15 minutes with different concentrations of the following agonists: adenosine diphosphate (ADP; Sigma-Aldrich), cross-linked collagen-related peptide (CRP-XL; Cambcol), and PAR 4-activating peptide (PAR4-AP, AYPGKF-NH_2_; AnaSpec) with phosphate-buffered saline (PBS) serving as control. Samples were stained with labeled antibodies (20 minutes) and fixed with 1% formaldehyde (10 minutes). Finally, erythrocytes were lysed (150 mM NH_4_Cl, 10 mM KHCO_3_, 0.1 mM Na_2_EDTA, pH 7.4; 10 minutes), and cells were pelleted (600 × *g*, 5 minutes) and resuspended in PBS.

### 2.5 Flow cytometry and gating strategy

Platelets were identified using anti–CD41-BV421 antibody (1:100; MWReg30) and examined for platelet degranulation and integrin activation (Supplementary Figure S1) using the following antibodies: anti–CD62P-PE-Cy7 (1:200; RMP-1), anti–CD63-PerCP-Cy5.5 (1:100; NVG-2), anti–CD40L-APC (1:25; MR1; all from BioLegend), and JON/A-PE recognizing activated GPIIb/IIIa (1:20; Emfret Analytics). Samples were measured using CytoFLEX S flow cytometer and analyzed with the CytExpert 2.4 software (both from Beckman Coulter). Data were quantified as the percentage of positive platelets or mean fluorescence intensity of all platelets for each marker.

### 2.6 Statistical analysis

Statistical analyses and data presentation were performed with GraphPad Prism 8 software. Samples were measured in duplicate, and mean values were used for statistical analyses. Results are reported in boxplots with whiskers showing median, quartiles, minimum, and maximum values; each symbol represents 1 animal. All the reported plots are representative of at least 6 independent experiments. Results were analyzed for Gaussian distribution using the Shapiro–Wilk test, and differences between datasets were evaluated by 2-way analysis of variance with Geisser–Greenhouse correction and uncorrected Fisher least significant difference test for multiple comparisons, with *P* values < .05 being considered statistically significant. Matched analyses were performed for data derived from 1 donor animal. Different *P* values are indicated as ∗*P* < .05, ∗∗*P* < .01, ∗∗∗*P* < .001, and ∗∗∗∗*P* < .0001.

## 3 Results

### 3.1 Blood collection site primarily influences platelet responsiveness to CRP-XL and PAR4-AP, but not to ADP

**Figure 1:**
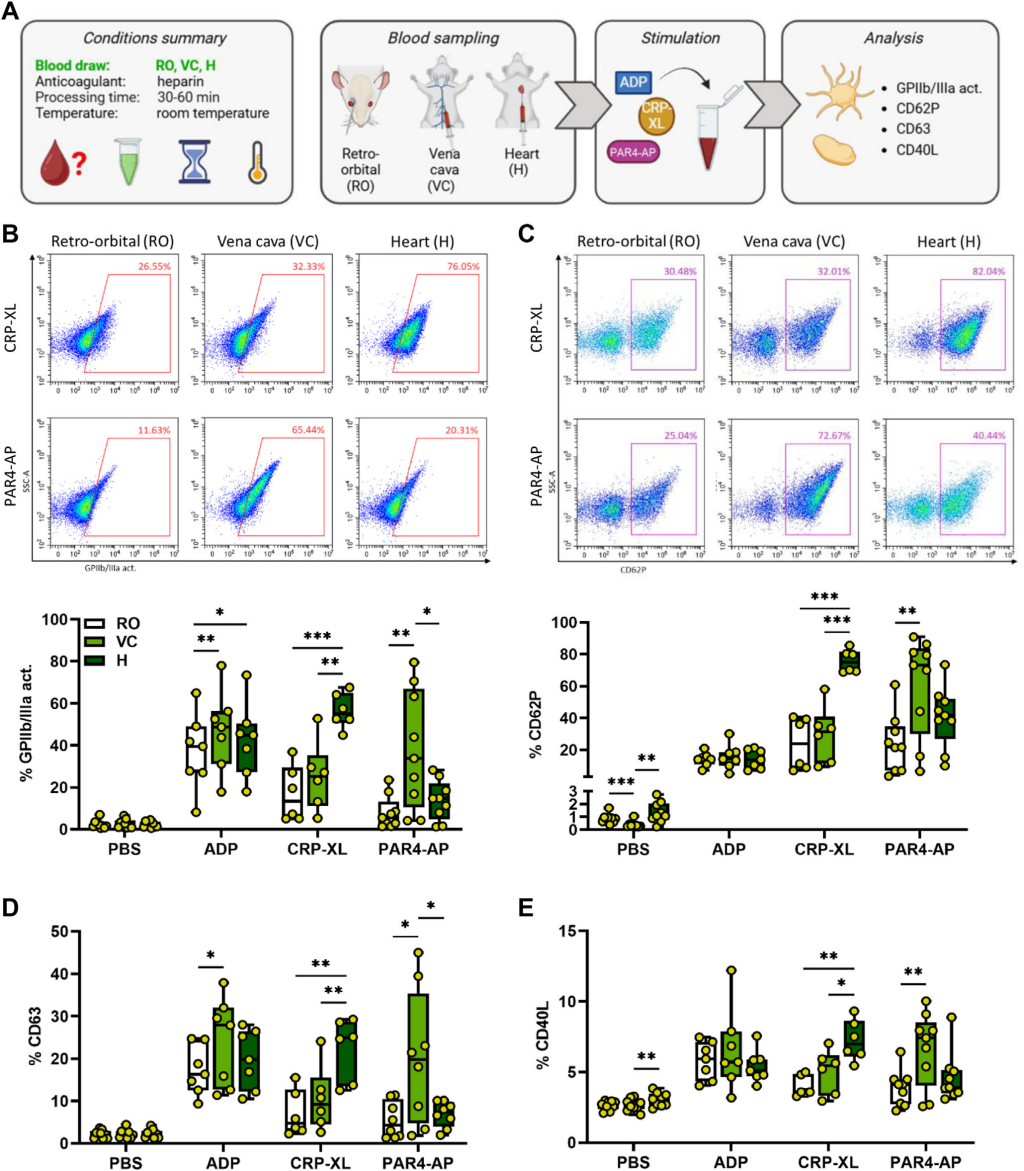
Blood collection method influences platelet glycoprotein (GP) IIb/IIIa activation and degranulation. (A) Experimental procedure: heparinized whole blood was collected from C57BL/6J mice using 3 different approaches: retro-orbital (RO), vena cava (VC), and heart (H) puncture. Samples were stimulated with phosphate-buffered saline (PBS) or with the following agonists: adenosine diphosphate (ADP; 250 μM), cross-linked collagen-related peptide (CRP-XL; 200 ng/mL), and protease-activated receptor 4-activating peptide (PAR4-AP; 70 μM). The stimulation was conducted for 15 minutes at room temperature. Flow cytometry was performed to analyze (B) GPIIb/IIIa activation and the expression of granule secretion markers (C) CD62P, (D) CD63, and (E) CD40L. Representative dot plots are shown in B and C. *N* = 6 to 9. ∗*P* < .05, ∗∗*P* < .01, ∗∗∗*P* < .001. Act., activity.

To analyze the impact of commonly used blood sampling procedures on murine platelet function, blood was drawn via 3 different approaches, RO, VC, and H, and stimulated with a panel of selected agonists (ADP, CRP-XL, and PAR4-AP; Figure 1A). Of note, as anesthesia with ketamine/xylazine reduces blood flow and promotes coagulation in mice relative to isoflurane [27,28], we performed these experiments with ketamine/xylazine only. Basal CD62P expression was reduced in VC-derived platelets compared with RO- and H-derived platelets, while the expression of activated GPIIb/IIIa and the degranulation markers CD63 and CD40L was not affected in basal conditions (Figure 1B–E). Notably, we observed agonist-specific modulation of platelet function by different collection methods, with ADP responses being least affected, whereas responses to CRP-XL and PAR4-AP were differently regulated.

Compared with RO sampling, VC-derived platelets showed significantly increased levels of active GPIIb/IIIa, CD62P, CD63, and CD40L in response to PAR4-AP stimulation. However, platelet responsiveness to CRP-XL did not differ at all between RO- and VC-sampling and platelet activation upon ADP stimulation was only partially regulated, with moderately higher levels of active GPIIb/IIIa and CD63 in VC-derived platelets relative to RO-derived platelets (Figure 1B–E). In contrast, platelets obtained by H displayed a distinctly different response profile. They closely resembled RO-derived platelets in their reactivity toward ADP or PAR4-AP, with only very minor changes in ADP-induced GPIIb/IIIa activation (Figure 1B). However, H-derived platelets were profoundly hyper-responsive to CRP-XL relative to both RO- and VC-derived platelets, as observed in all 4 activation markers assessed (Figure 1B–E).

Intriguingly, when the incubation steps were carried out at 37 °C instead of RT, the observed differences between RO-, VC-, and H-derived platelets were less pronounced, with VC-derived platelets showing moderately higher PAR4-AP responsiveness, while H-derived platelets displayed only slightly higher degranulation upon CRP-XL (Supplementary Figure S2A–E).

Overall, blood draw procedure had little to no effect on platelet activation by ADP, but VC-derived platelets were hyper-responsive to PAR4-AP, whereas H-derived platelets were hyper-responsive to CRP-XL. Blood sampling effects were less pronounced when *in vitro* stimulation was performed at 37 °C rather than at RT.

### 3.2 Calcium-chelating anticoagulants dampen platelet activation by PAR4-AP but not by CRP-XL

**Figure 2:**
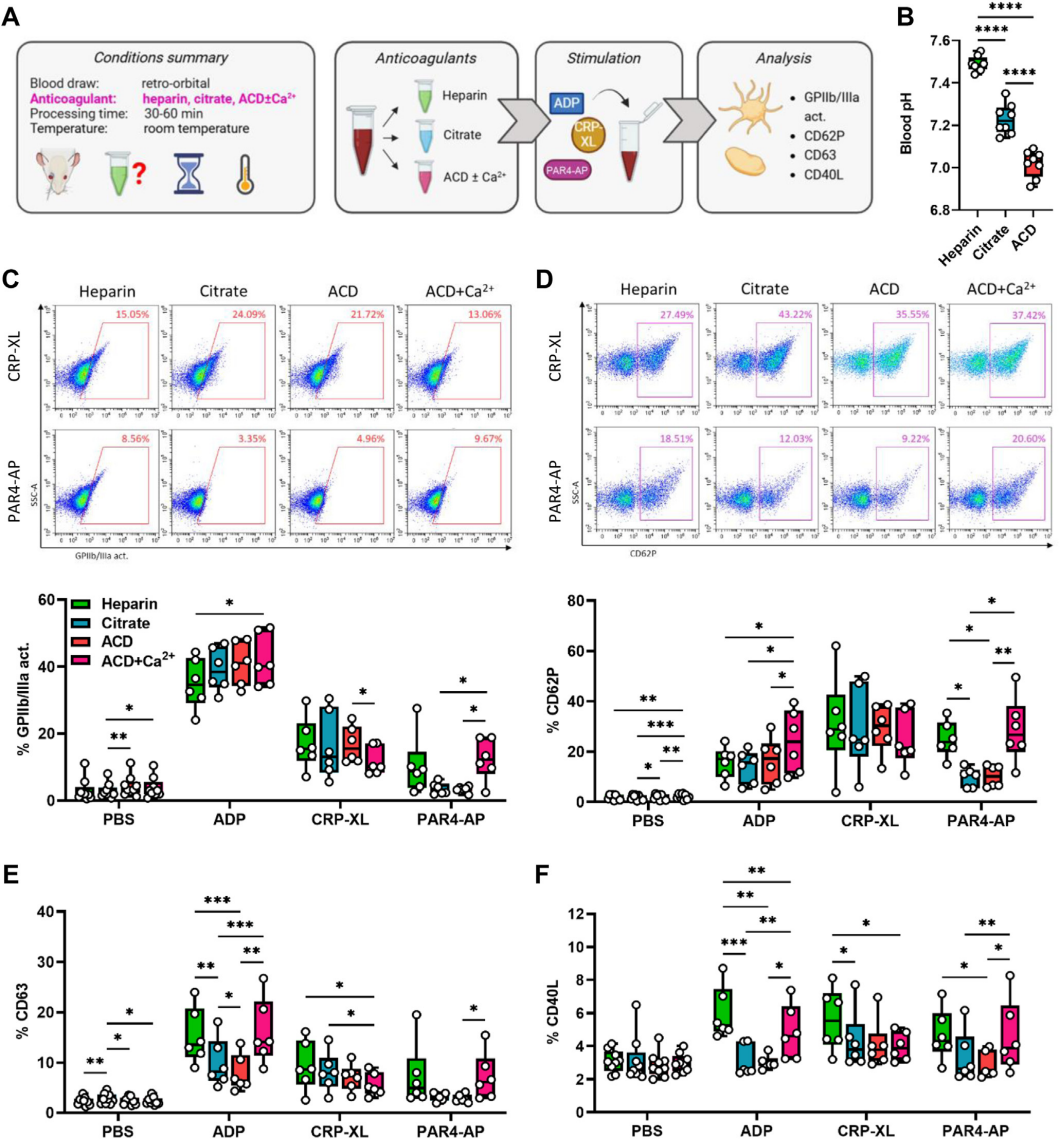
Calcium (Ca^2+^)-chelating anticoagulants impair platelet activation and degranulation induced by adenosine diphosphate (ADP) and protease-activated receptor 4-activating peptide (PAR4-AP) but not by cross-linked collagen-related peptide (CRP-XL). (A) Experimental procedure: whole blood was collected from C57BL/6J mice by retro-orbital puncture and anticoagulated with heparin, citrate, acid-citrate-dextrose (ACD), or ACD supplemented with 2 mM Ca^2+^ (ACD + Ca^2+^). Samples were stimulated with phosphate-buffered saline (PBS) or with the following agonists: ADP (250 μM), CRP-XL (80 ng/mL), and PAR4-AP (70 μM) for 15 minutes at room temperature. (B) Whole blood pH in heparin-, citrate-, and ACD-anticoagulated blood samples was measured before further processing. (C–F) Platelet activation was measured by flow cytometry, analyzing (C) glycoprotein (GP) IIb/IIIa activation and the expression of the degranulation markers (D) CD62P, (E) CD63, and (F) CD40L. Representative flow cytometry dot plots are shown in C and D. *N* = 6 to 9. ∗*P* < .05, ∗∗*P* < .01, ∗∗∗*P* < .001. Act., activity.

Next, we investigated how anticoagulants affect murine platelet responsiveness. Due to the widespread use of heparinized capillaries for drawing rodent blood, we decided to take this perpetual presence of heparin into account, using heparin alone or in combination with the calcium chelators citrate or ACD. In addition, we evaluated the effect of recalcification by supplementing ACD-anticoagulated blood with CaCl_2_ to a final concentration of 2 mM (Figure 2A). As expected, addition of citrate or ACD acidified the blood to approximately pH 7.2 or pH 7.0, respectively (Figure 2B). None of the samples showed any signs of preactivation, with only slight variations in basal GPIIb/IIIa activation and CD62P and CD63 expression (Figure 2C–E). Again, anticoagulation and recalcification differently affected agonist responses.

Presence of citrate or ACD almost abolished PAR4-AP–induced platelet activation, affecting all 4 evaluated activation markers, which was restored upon addition of calcium (Figure 2C–F), demonstrating that PAR4-AP–induced platelet activation critically depends on extracellular calcium. Interestingly, while ADP-induced CD63 and CD40L expression was also calcium-dependent (Figure 2E, F), citrate or ACD did not affect ADP-induced GPIIb/IIIa activation or CD62P expression, though recalcification levels were slightly raised (Figure 2C, D). In contrast, platelet activation upon CRP-XL was independent of calcium-chelating anticoagulation. Surprisingly, recalcification even dampened platelet responses to CRP-XL, though effects were very minor. Similar results were obtained when platelets were stimulated at 37 °C. Of note, at 37 °C, calcium-chelating anticoagulants fostered ADP-induced GPIIb/IIIa activation (Supplementary Figure S3A–E).

Overall, platelet activation by CRP-XL appears to be independent of anticoagulants, whereas PAR4-AP responses are strongly impaired by citrate or ACD and restored by recalcification. The effect of anticoagulation on ADP is dichotomous with CD63 and CD40L, but not CD62P or GPIIb/IIIa, exhibiting calcium dependence.

### 3.3 Modulation of platelet responsiveness by delayed processing occurs within the first 60 minutes

**Figure 3:**
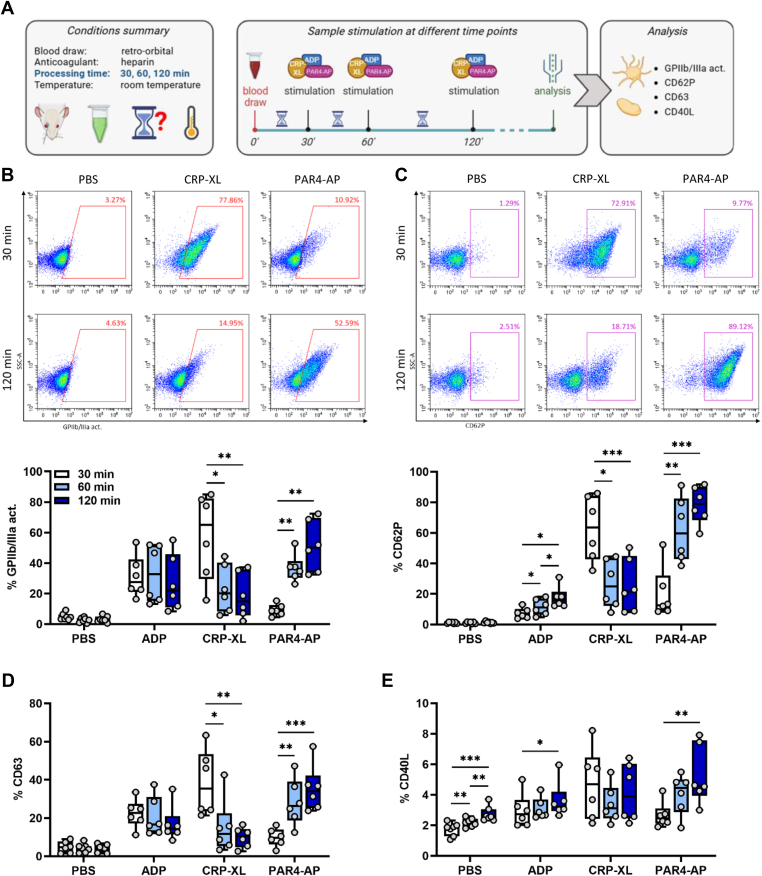
Processing time affects platelet glycoprotein (GP) IIb/IIIa activation and degranulation. (A) Experimental procedure: heparinized whole blood was collected from C57BL/6J mice by retro-orbital sampling and stimulated 30, 60, or 120 minutes after sampling with phosphate-buffered saline (PBS), adenosine diphosphate (ADP; 250 μM), cross-linked collagen-related peptide (CRP-XL; 200 ng/mL), and protease-activated receptor 4-activating peptide (PAR4-AP; 85 μM) for 15 minutes at room temperature. Flow cytometry was performed to analyze (B) GPIIb/IIIa activation and the expression of the degranulation markers (C) CD62P, (D) CD63, and (E) CD40L. Representative dot plots are shown in B and C. *N* = 6 to 7. ∗*P* < .05, ∗∗*P* < .01, ∗∗∗*P* < .001. Act., activity.

In murine models, immediate analysis of platelet function is often unfeasible due to logistical restraints such as harvesting samples of multiple animals or infrastructural limitations, resulting in delayed blood processing. During this delay, stasis and oxidation processes may alter platelet reactivity. To assess how the time between sample collection and processing affects platelet response, different agonist responses were determined at 30, 60, or 120 minutes after collection (Figure 3A). Importantly, processing delay did not significantly affect basal levels of GPIIb/IIIa activation (Figure 3B) or the expression of CD62P (Figure 3C) and CD63 (Figure 3D), though surface CD40L exhibited a slight increase over time (Figure 3E). Again, the effects of processing delays on platelet responsiveness were agonist-specific.

A delay of 30 to 60 minutes resulted in a marked reduction in CRP-XL–induced GPIIb/IIIa activation and degranulation marker expression (CD62P and CD63), but conversely enhanced these markers in response to PAR4-AP. Further extending the delay to 120 minutes did not significantly exacerbate CRP-XL hypo-responsiveness and PAR4-AP hyper-responsiveness any further.

In contrast, ADP-induced platelet responses were quite stable over prolonged delays, with primarily minor increases in CD62P expression over time (Figure 3B–E).

Overall, while processing delays minimally influenced platelet responsiveness to ADP, they severely diminished responses to CRP-XL and significantly amplified sensitivity to PAR4-AP. Notably, these changes in platelet reactivity were predominantly established within the first 60 minutes after blood collection, with further delays having minimal additional effects.

### 3.4 Ambient temperature fosters platelet responsiveness to ADP and CRP-XL compared with 37 °C

Given that hypothermic mice exhibit augmented vessel attachment and thrombotic occlusion [9,29], we investigated *in vitro* modulation of murine platelet function by temperature. Specifically, the possible influence of temperature on platelet activation was analyzed by stimulating whole blood samples at RT or 37 °C.

**Figure 4:**
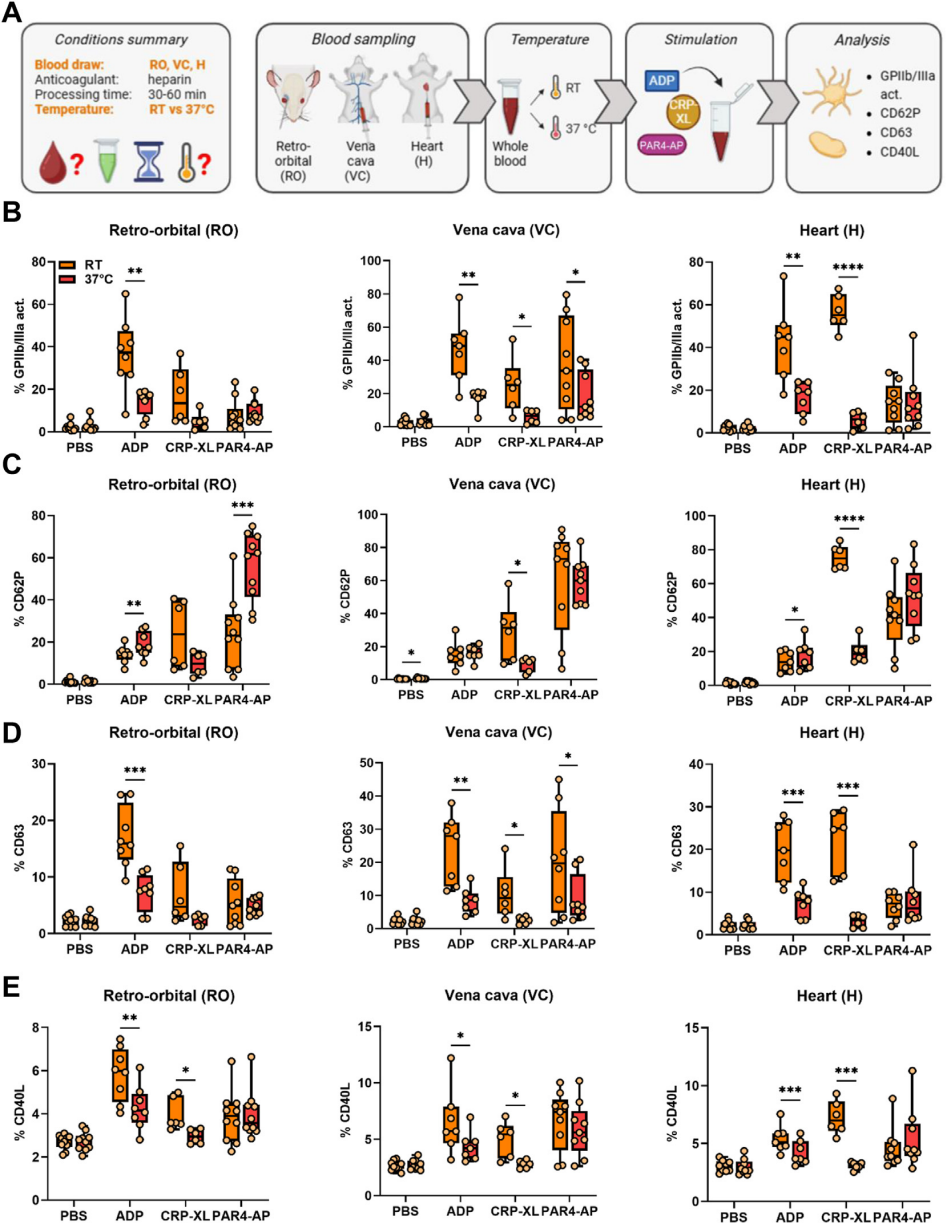
Experimental temperature modulates platelet activation with a similar trend in different blood sampling conditions. (A) Experimental procedure: whole blood was collected and processed as described in Figure 1. Sample stimulation was performed at room temperature (RT) or 37 °C. Platelet activation was measured by flow cytometry, analyzing (B) glycoprotein (GP) IIb/IIIa activation and the expression of granule secretion markers (C) CD62P, (D) CD63, and (E) CD40L. *N* = 6 to 9. ∗*P* < .05, ∗∗*P* < .01, ∗∗∗*P* < .001, ∗∗∗∗*P* < .0001. Act., activity. ADP, adenosine diphosphate; CRP-XL, cross-linked collagen-related peptide; H, heart; PAR4-AP, protease-activated receptor 4-activating peptide; PBS: phosphate-buffered saline; RO, retro-orbital; VC, vena cava.

This strategy was adopted for the treatment of samples collected by different sampling methods (RO, VC, and H; Figure 4A). No platelet preactivation was observed either at RT or 37 °C (Figure 4B–E). Overall, differences in incubation temperature similarly regulated ADP- and CRP-XL–induced platelet activation. GPIIb/IIIa activation as well as expression of CD63 and CD40L were significantly impaired in samples stimulated at 37 °C compared with those incubated at RT. This observation was independent of the blood sampling technique, though differences were strongest in H-derived platelets (Figure 4B, D, E). Conversely, while 37 °C also diminished CRP-XL–induced CD62P expression, ADP-induced CD62P expression was slightly but significantly enhanced. Of note, this difference was only observed in RO- and H-derived platelets, but not in VC-derived ones (Figure 4C). Temperature-mediated differences were less pronounced upon PAR4-AP stimulation. In VC-derived platelets, both GPIIb/IIIa activation and CD63 induced by PAR4-AP were dampened at 37 °C (Figure 4B, D), whereas CD62P expression was elevated in RO-derived platelets (Figure 4C).

We further adopted our protocol to investigate the potential interaction between temperature and anticoagulants (Supplementary Figure S4A), but the overall effects of temperature on platelet activation proved to be independent of anticoagulants (Supplementary Figure S4B–E).

Generally, platelet incubation at 37 °C, relative to RT, markedly decreased levels of active GPIIb/IIIa, CD63, and CD40L induced by ADP or CRP-XL, but not by PAR4-AP. However, modulation of CD62P by temperature was more multifaceted, as 37 °C inhibited CRP-XL–induced CD62P but augmented ADP- and PAR4-AP–induced CD62P.

## 4 Discussion

With this study, we aim to raise awareness and increase the understanding of how procedural variations—common across research groups, experiments, or even among individual samples—modulate murine platelet responsiveness *in vitro*. While microenvironmental effects on human platelet function are well-documented [[4], [5], [6], [7], [8], [9], [10], [11]], the sensitivity of murine platelets to protocol variables remains understudied. Given the widespread use of murine models to study platelet (patho-) physiology, this gap in understanding poses challenges in designing and interpreting experimental results.

Using flow cytometry to assess degranulation markers (CD62P, CD63, and CD40L) and GPIIb/IIIa activation, we demonstrate that key procedural steps—blood draw techniques, choice of anticoagulant, processing delays, and temperature—significantly influence murine platelet activation in an agonist-specific manner.

Blood sampling sites are often chosen based on practical reasons, eg, guidelines, blood volume, reproducibility, and personnel expertise. While Grill et al. [30] reported comparable basal platelet activation across RO, VC, and H blood samples, we observed slightly elevated basal CD62P expression upon H puncture, consistent with anecdotal reports [31]. ADP sensitivity remained largely unaffected, but platelet responses to CRP-XL and PAR4-AP varied significantly by draw site. Human platelet function is largely unaffected by sampling site [[32], [33], [34], [35]], but the invasive nature of murine blood draws may contribute to differences in platelet reactivity. RO sampling can cause tissue trauma and contamination with tissue fluid [36], while H puncture may alter the local hemostatic environment due to the tissue factor-rich nature of the H [37]. We found slightly lower maximal blood volumes obtainable from terminal RO sampling (maximum: 800 μL; mean ± SD: 540 ± 110 μL) compared with H or VC (maximum: 1000 μL; mean ± SD: 790 ± 150 μL). Volume reproducibility depends on factors like sex, weight, health status, anesthesia, and operator skill. Although all methods can be effective with experience, anatomical factors (eg, excessive abdominal fat or microphthalmia) may complicate sampling. Of note, RO sampling allows nonterminal blood collection under short-term inhalation anesthesia, enabling serial measurements—ideal for flow cytometric analyses, which require only small volumes.

Anticoagulation critically affects platelet function. Compared with heparin, citrate and ACD almost abolished platelet activation by PAR4-AP but had little effect on ADP or CRP-XL responses, suggesting that extracellular calcium is essential for PAR4 but not GPVI signaling in murine platelets. While data on murine platelets remain limited, one study reported lower basal GPIIb/IIIa activation in citrate vs heparin [30]. This is supported by extensive human studies demonstrating that citrate and ACD better preserve biomechanical properties, reduce spontaneous activation, and decrease aggregation in response to various agonists, including ADP, collagen, and thrombin receptor-activating peptide 6 [5,[38], [39], [40]]. This contrasts with the agonist-specific effects observed in mice, possibly due to weaker priming or activating effects of heparin and its derivatives involving outside-in signaling [41] in murine platelets, which are generally less responsive to agonists and mechanical stress than human platelets. We found no differences in platelet function between citrate- and ACD-anticoagulated murine blood, despite pH differences known to regulate platelet activation *in vitro* and hemostasis *in vivo* [7,11,[42], [43], [44]]. Similarly, human platelets show comparable GPIIb/IIIa activation and deformability, though ACD better prevents spontaneous degranulation and lysis [38,40,45], making ACD preferable for prolonged storage.

In murine models, simultaneous analysis of multiple animals enhances efficiency but introduces processing delays. We observed stable basal platelet activation for up to 120 minutes, which is similar to human platelets [34]. Spontaneous degranulation increases thereafter, but can be diminished by optimal choice of anticoagulants such as ACD or citrate-theophylline-adenosine-dipyridamole [38]. Even brief delays of only 60 minutes significantly affected murine platelet responses to CRP-XL and PAR4-AP, with limited further impact over time. These effects differ from those of human platelets, which also exhibit agonist-specific effects of processing delays but involve different agonists [5,39,46]. For instance, GPVI and PAR signaling pathways show opposite trends in murine and human platelets, suggesting fundamental differences in platelet biology between these species. Previous studies [5,39,46] and our results suggest that delay effects are most pronounced within the first hour. If immediate or staggered analysis is not feasible, storing samples for at least 60 minutes may reduce variability. To prevent bias in large mouse cohorts, blood collection order should be randomized.

Temperature also strongly modulates murine platelet function, with increased sensitivity to ADP and CRP-XL at RT compared with 37 °C. This is in line with previous observations demonstrating that hypothermic conditions, spanning a temperature range of 20 to 35 °C, enhance platelet activation across multiple agonists [11,[47], [48], [49], [50], [51], [52], [53]], suggesting a shared mechanism. Hypothermia inhibits CD39-mediated ADP degradation [9] and promotes actin assembly and reorganization, thereby sustaining platelet activation and shape change [54]. *In vivo,* hypothermic mice exhibit augmented platelet activation, accelerated thrombus formation, and stability upon FeCl_3_ challenge [9,29]. We and others [11,29,51] observed that hypothermia predominantly increased GPIIb/IIIa activation over CD62P exposure, which pivotally facilitates platelet-leukocyte interactions [55]. As previously suggested, this seemingly counterintuitive platelet characteristic may reflect physiologic temperature differences between body core and surface, where vascular injuries are most likely, whereas platelets may be evolutionarily optimized to remain placid while passing through central vasculature [54]. Accordingly, optimal assay temperature may differ for prothrombotic and immunomodulatory platelet functions. Notably, effects of hypothermia are transient [29,53], and stimulation but not preincubation temperature determines platelet responsiveness [6], facilitating assay design.

Of note, our study has several limitations. First, we exclusively utilized C57BL/6 mice, which may respond differently to microenvironmental fluctuations than other strains. Additionally, we conducted our experiments in whole blood, which preserves physiological conditions and avoids artifacts from cell isolation, eg, due to receptor desensitization or shedding [56,57], but limits mechanistic insight into contributions of, eg, plasma factors or other blood cells. Due to unclear leukocyte sensitivity to ADP and/or PAR4-AP, off-target activation cannot be excluded. Future studies using platelet-rich plasma and/or isolated platelets are necessary to separate direct from indirect effects. We also tested a limited set of stimuli and readouts, so other responses, especially immunomodulatory platelet functions, may be regulated differently. Platelet activation was primarily assessed as the percentage gated, which is sensitive to weak/moderate stimulation, but less accurate for strong activation [6]. To address this, we confirmed key findings using mean fluorescence intensity measurements (Supplementary Figure S5).

## 5 Conclusion

Our findings highlight the importance of considering agonist-specific responses in murine platelet function studies. Procedural variations, such as blood sampling technique, anticoagulation, processing delays, and temperature, can significantly affect activation, necessitating protocol optimization to minimize bias and ensure reproducibility.
