# Supplementary material for: Impact of sampling technique, anticoagulant, processing delay, and temperature on murine platelet function in whole blood
Source: Res Pract Thromb Haemost. 2025 May 8;9(4):102883. doi: 10.1016/j.rpth.2025.102883 (PMC12166811; doi:10.1016/j.rpth.2025.102883)
Supplement: Supplementary Figures S1-S5 [file mmc1.pdf]

## SUPPLEMENTARY FIGURES

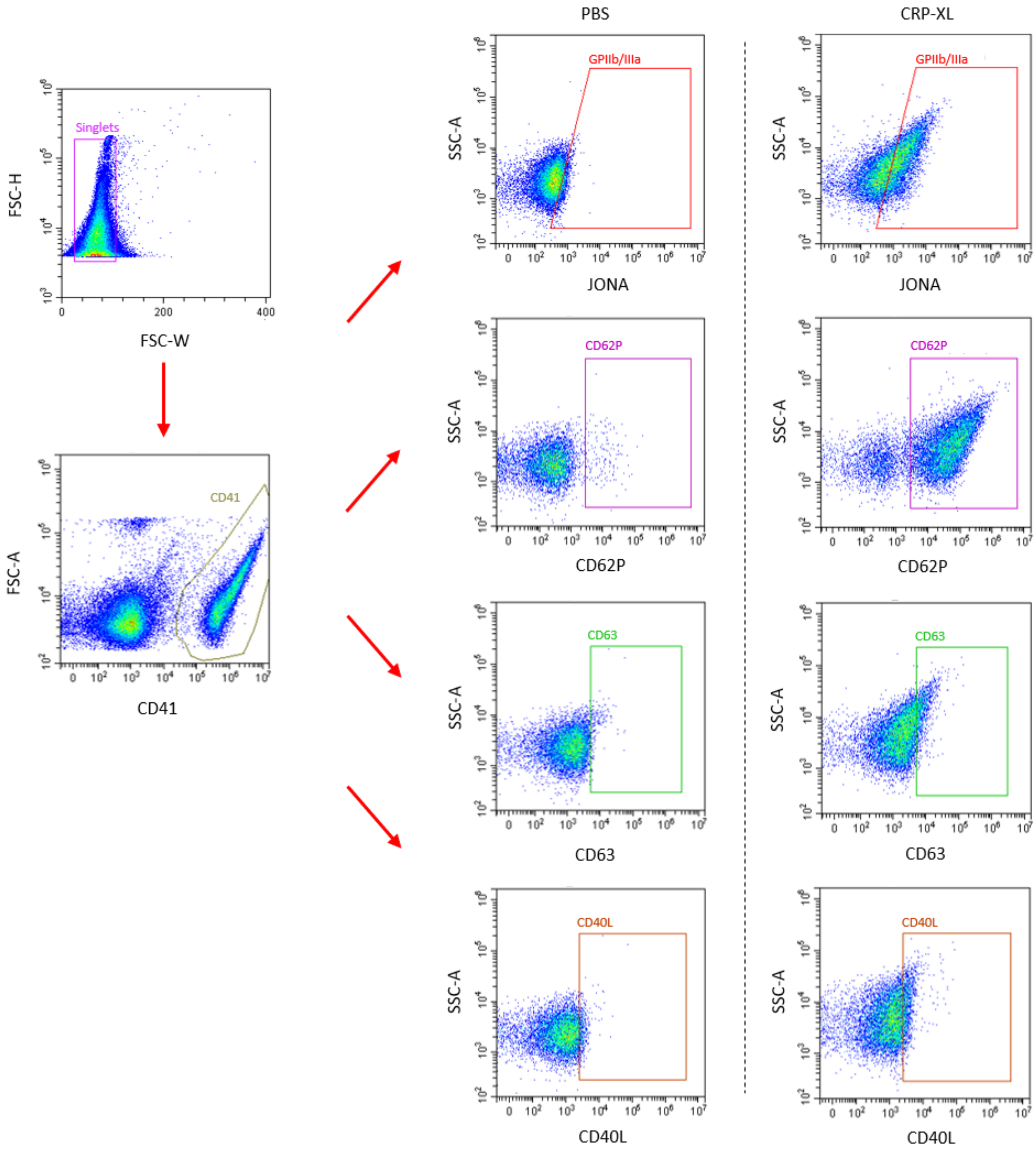


**Supplementary Figure S1: Gating strategy to evaluate platelet activation.** Single cells (singlets) were selected by evaluating forward scatter parameters FSC-H vs FSC-W. Platelets were identified by analyzing the events positive for the platelet-specific integrin CD41. Platelet activation was further measured by analyzing the expression of CD62P, CD63, CD40L and the activated form of GPIIb/IIIa recognized by JON/A antibody and evaluated as % gated or mean fluorescence intensity (MFI) of all platelets. Plots show a heart puncture-derived sample stimulated at room temperature with either PBS or CRP-XL.

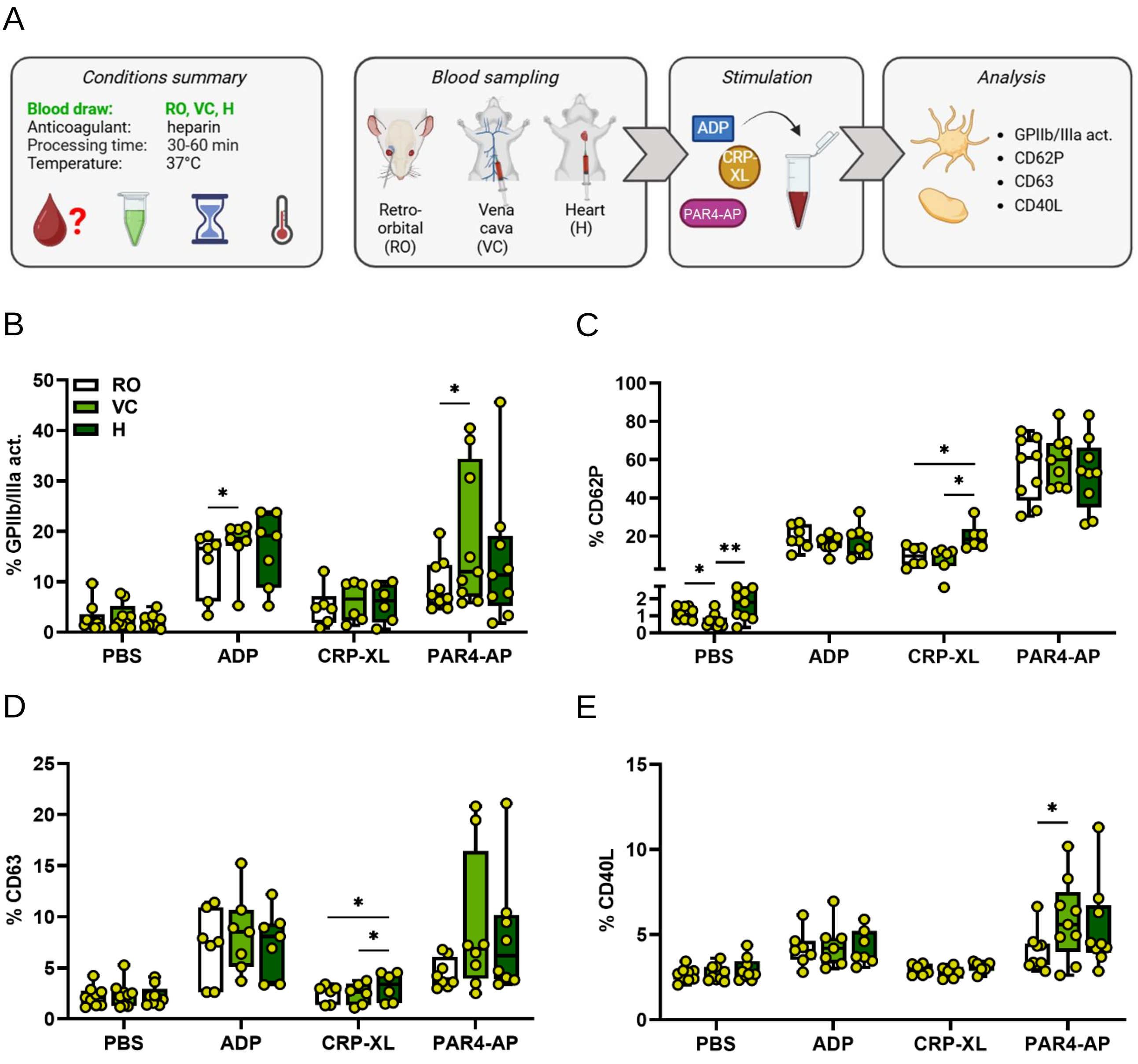


**Supplementary Figure S2: Blood collection method weakly affects platelet activation at 37°C.** (A) Whole blood was collected and processed as described in Fig. 1. Sample stimulation was performed at 37°C. Platelet activation was measured by flow cytometry, analyzing (B) GPIIb/IIIa activation and the expression of the degranulation markers (C) CD62P, (D) CD63 and (E) CD40L. n=6-9. *p<0.05, **p<0.01.

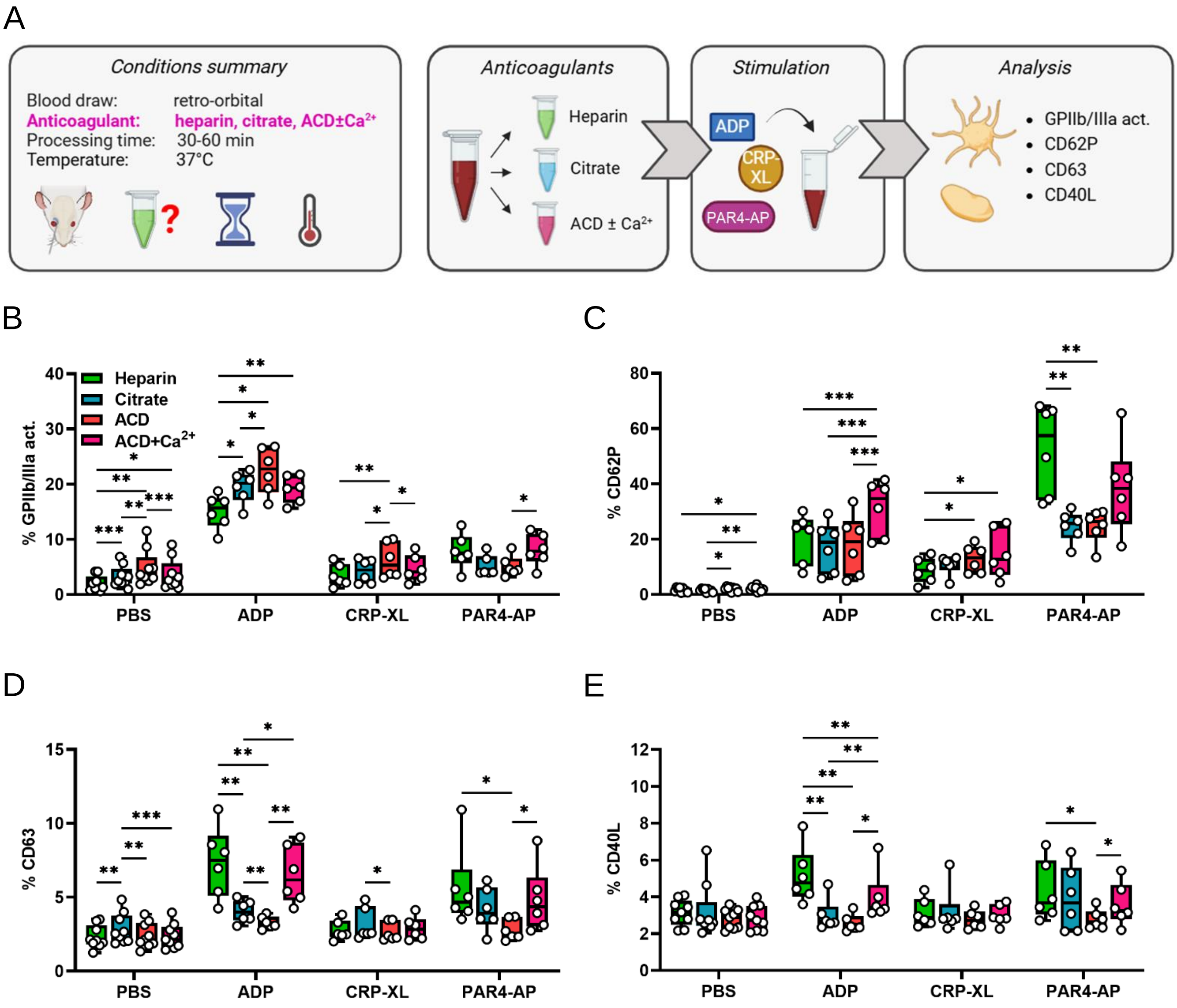


**Supplementary Figure S3: The type of anticoagulant primarily influences platelet activation induced by ADP and PAR4-AP, but not CRP-XL, upon stimulation at 37°C.** (A) Whole blood was collected and processed as described in Fig. 2. Sample stimulation was performed at 37°C. Platelet activation was measured by flow cytometry, analyzing (B) GPIIb/IIIa activation and the expression of granule secretion markers (C) CD62P, (D) CD63 and (E) CD40L. n=6-9. *p<0.05, **p<0.01, and ***p<0.001.

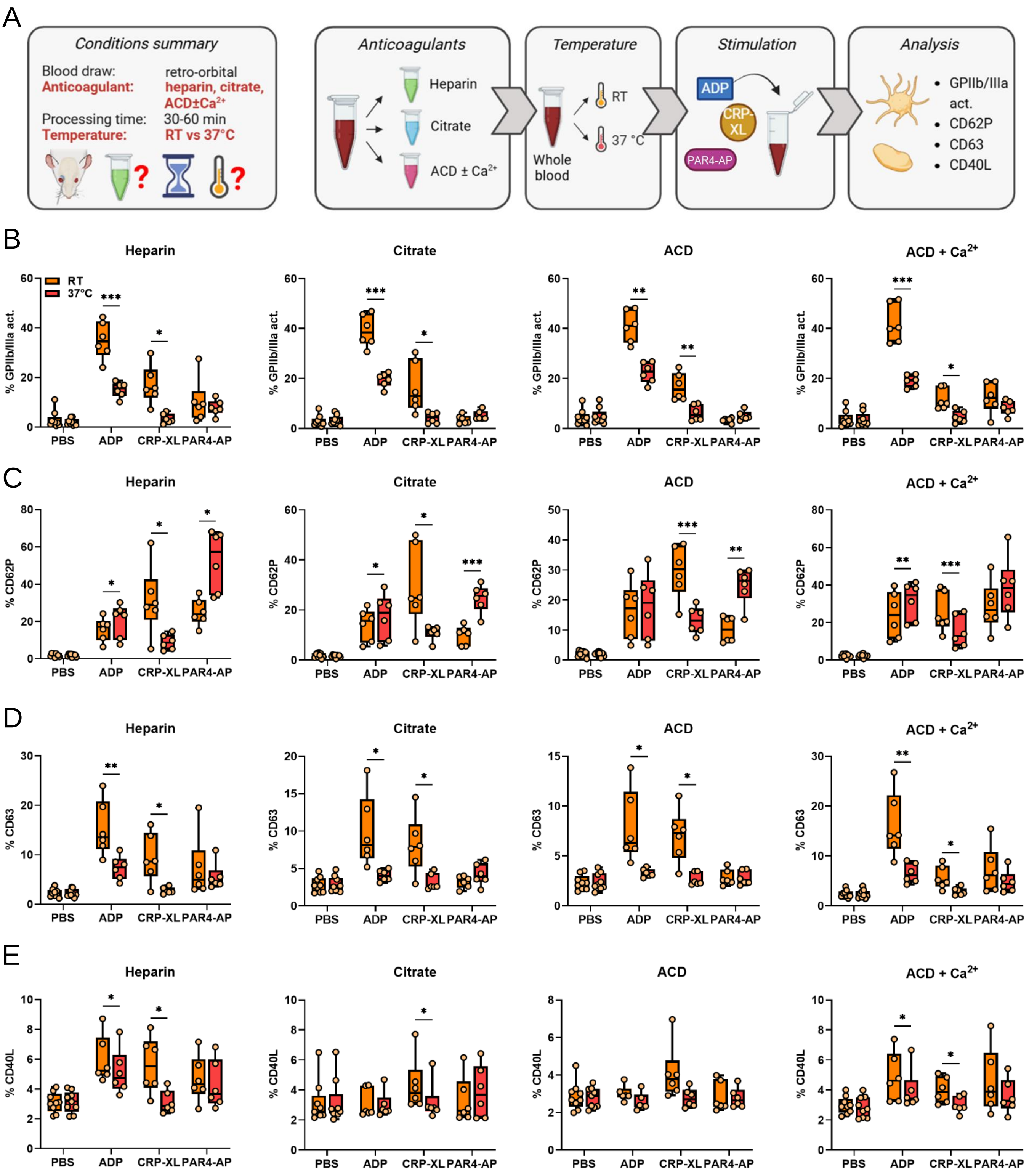


**Supplementary Figure S4: Experimental temperature modulates platelet activation regardless of the type of anticoagulant used.** (A) Experimental procedure: whole blood was collected and processed as described in Fig. 2. Sample stimulation was performed at room temperature (RT, orange), or 37°C (red). Platelet activation was measured by flow cytometry, analyzing (B) GPIIb/IIIa activation and the expression of the degranulation markers (C) CD62P, (D) CD63 and (E) CD40L. n=6-9. *p<0.05, **p<0.01, and ***p<0.001.

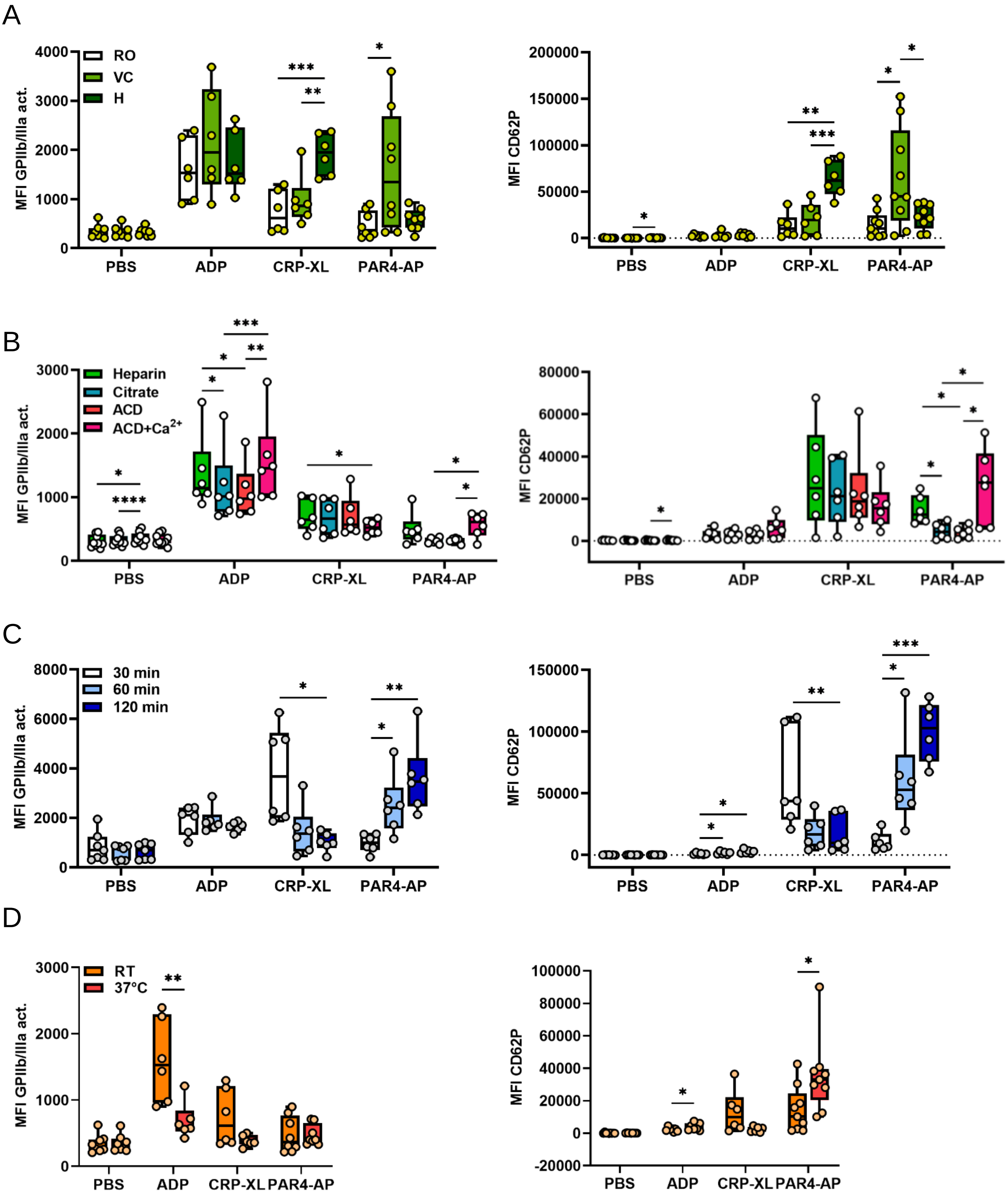


**Supplementary Figure S5: Impact of blood draw technique, anticoagulant, processing delay and temperature on surface levels of CD62P and activated GPIIb/IIIa.** Murine whole blood was obtained and processed as described in (A) Figure 1, (B) Figure 2, (C) Figure 3 or (D) Figure 4 (retro-orbital blood draw). Basal and agonist-induced platelet activation was evaluated by quantifying the mean fluorescence intensity (MFI) of activated GPIIb/IIIa (left) and CD62P (right) of all CD41-positive platelets. n=6-9. *p<0.05, **p<0.01, ***p<0.001, and ****p<0.0001.
